# Correlation Study on the Expression of INSR, IRS-1, and PD-L1 in Nonsmall Cell Lung Cancer

**DOI:** 10.1155/2022/5233222

**Published:** 2022-10-04

**Authors:** Ma Ting, Yu-e Miao, Feng-xiu Yu, Guang-cai Luo, Xin Xu, Li-xia Xiao, Guo-qing Zhang, Jin Chang

**Affiliations:** ^1^Shandong First Medical University & Shandong Academy of Medical Sciences, Tai'an, 271000 Shandong Province, China; ^2^The Second Affiliated Hospital of Shandong First Medical University, Tai'an, 271000 Shandong Province, China; ^3^Department of Chemotherapy, The Second Affiliated Hospital of Shandong First Medical University, Tai'an 271000, Shandong Province, China; ^4^Basic Medical College, Shandong First Medical University and Shandong Academy of Medical Sciences, Tai'an, 271000 Shandong Province, China; ^5^Department of Radiation Oncology, The Second Affiliated Hospital of Shandong First Medical University, Tai'an 271000, Shandong Province, China

## Abstract

**Objective:**

To study the expression and correlation of insulin receptor (INSR), insulin receptor substrate-1 (IRS-1), and programmed cell death ligand-1 (PD-L1) in nonsmall cell lung cancer (NSCLC).

**Methods:**

45 lung cancer tissues and 30 adjacent normal tissues of NSCLC patients diagnosed in the Second Affiliated Hospital of Shandong First Medical University from June 2019 to August 2020 were selected. The expressions of INSR, IRS-1, and PD-L1 proteins in tumor tissues and adjacent tissues of NSCLC were detected by immunohistochemical staining.

**Results:**

The expression of INSR and IRS-1 in NSCLC was significantly higher than that in adjacent normal lung tissue (*P* < 0.05). INSR expression had statistical significance with the degree of pathological differentiation of nonsmall cell carcinoma (*P* = 0.031), but had no significant association with age, gender, pathological type, TNM stage, and lymph node metastasis status (*P* > 0.05). There was no significant correlation between IRS-1 positive expression and NSCLC patients' age, gender, pathological typing, degree of differentiation, TNM stage, and lymph node metastasis (*P* > 0.05). PD-L1 positive expression was correlated with lymph node metastasis of NSCLC (*P* = 0.028), while there was no significant correlation with gender, age, pathological type, TNM stage, and pathological differentiation degree of NSCLC patients (*P* > 0.05). Spearman correlation analysis showed that PD-L1 protein expression had a significant positive correlation with IRS-1 protein expression (*r* = 0.373), but was not correlated with the expression of INSR protein.

**Conclusion:**

IRS-1 may be involved in the regulation of PD-L1 expression and mediate the occurrence of tumor immune escape, which is expected to become a new target for NSCLC immunotherapy and provide new clinical evidence for immunosuppressive therapy.

## 1. Introduction

Lung cancer is a common malignant tumor in the world. According to the latest data released by the international agency for research on cancer in *CA:A Cancer Journal for Clinicians* [1], there were 2.207 million new lung cancer cases and 1.796 million reported deaths worldwide, accounting for 11.7% and 18.0% of the global cancer incidence rate and mortality, respectively. Nonsmall cell lung cancer (NSCLC) has decreased in incidence overall in the past decade in the United States but the incidence of stage I NSCLC has increased along with its prevalence [2], highlighting the need for research in this area. Surgical resection can be the first choice for the treatment of NSCLC. However, more than 70% of patients with NSCLC were often accompanied by tumor proliferation and metastasis and lost the best opportunity for surgery due to the limitations of early diagnosis [3]. According to a global cancer detection report, lung cancer patients in most countries had a 5-year survival rate of only 10-20% [4]. With the continuous development of cancer treatment methods and technologies, the situation has improved [5]. But the problems of drug resistance and immunotoxicity cannot be ignored. Therefore, seeking new treatment targets and exploring their related mechanisms in the occurrence and development of lung cancer are the current research focus and urgent problem to be solved. Therefore, seeking new treatment targets and exploring their related mechanisms in the occurrence and development of lung cancer are the current research focus and urgent problem to be solved.

INSR and IRS-1 can mediate tumorigenesis and development as key mediators in the insulin signal transduction pathway, but their specific biological roles have not been fully clarified. Zhang et al. [6] found that downregulation of INSR can inhibit tumor cell proliferation, angiogenesis, lymphangiogenesis, and metastasis. Kim et al. [7] studied the effect of INSR expression on the survival of patients with NSCLC for the first time and found that INSR expression could be used as an independent prognostic factor for OS and RFS in patients with surgically resected early NSCLC. INSR has been proved to be abnormally expressed in a variety of tumor tissues, such as breast cancer, lung cancer, and gastric cancer [7–9]. Li et al. [10] first found that overexpression of IRS-1 can promote the malignant transformation of mouse embryonic fibroblasts. Subsequently, Tanaka et al. [11] further confirmed that IRS-1 overexpression can induce malignant transformation and proliferation of NIH3T3 fibroblasts by activating ERK1/2 pathway under low serum conditions. In addition, fibroblasts transfected with IRS can form colonies in soft agar, which is also highly tumorigenic when injected into nude mice. As a transmembrane protein, PD-L1 is considered to be an inhibitor of immune responses. It can combine with PD-1 to mediate the occurrence of tumor immune evasion [12]. With the approval of immune checkpoint inhibitors (ICIS), the treatment of immune checkpoints based on regulating PD-1/PD-L1 signaling pathway provides more options for the treatment of patients with advanced NSCLC [13, 14]. Gene expression studies indicate that PD-L1 expression is a biomarker of improved survival and response to immune checkpoint blockade therapy [15, 16]. IRS-1 expression is also associated with prognosis [17].

However, there are few reports on the correlation between INSR, IRS-1, and PD-L1 in NSCLC patients. Therefore, it is of great significance to explore the correlation between the expression levels of INSR, IRS-1, and PD-L1 and reveal the potential mechanism of insulin pathway-related receptors in the occurrence and development of lung cancer.

## 2. Materials and Methods

### 2.1. Tissue Samples

45 patients with NSCLC diagnosed in the Second Affiliated Hospital of Shandong First Medical University from June 2019 to August 2021 were selected. The 45 patients with nonsmall cell lung cancer, including 26 males and 19 females, did not receive radiotherapy, chemotherapy, and other antitumor treatments before surgery. The clinical data of all patients included in the experiment were complete, and the postoperative pathology had confirmed NSCLC. The TNM stage was determined according to the 2018 AJCC staging guidelines. This experiment has been approved by the medical ethics committee of our hospital before it is started.

### 2.2. Immunohistochemistry

The expression of INSR, IRS-1, and PD-L1 in 45 NSCLC tissues and 30 adjacent normal tissues were detected by immunohistochemistry (IHC). The results of immunohistochemical staining were determined by two pathologists using semiquantitative analysis method. The positive staining of INSR was localized in the cell membrane and cytoplasm, while the positive staining of IRS-1 was localized in the cytoplasm and nucleus. Observed the staining intensity and count the proportion of positive cells under a high power microscope. The intensity of the immunostaining was scored as follows: (a) no staining, 0; (b) pale yellow, 1; (c) brown-yellow, 2; and (d) brown, 3. The percentage of positive tumor cells was scored as (a) <5%, 0; (b) 5-25%, 1; (c) 26-50%, 2; (d) 51-75%, 3; and (e) >75%, 4. The two scores were multiplied to produce a weighted score for each case. Cases with weighted scores of more than 3 were defined as positive; otherwise; they were defined as negative. The positive staining of PD-L1 was mainly localized in the cell membrane, which was scored according to the proportion of tumor pigmented cells (TPS Score). When TPS ≥ 1%, it was defined as positive, otherwise, it was defined as negative.

### 2.3. Statistical Analysis

SPSS26.0 statistical software was used for data processing, and the comparison between positive expression rate, expression of each indicator, and clinicopathological data among groups was performed by chi-square test; the correlation between INSR, IRS-1, and PD-L1 expression was tested by Spearman rank correlation analysis. When the default *P* < 0.05, the difference was considered statistically significant.

## 3. Results

### 3.1. Expression of INSR and IRS-1 in NSCLC and Adjacent Normal Tissues

**Figure 1:**
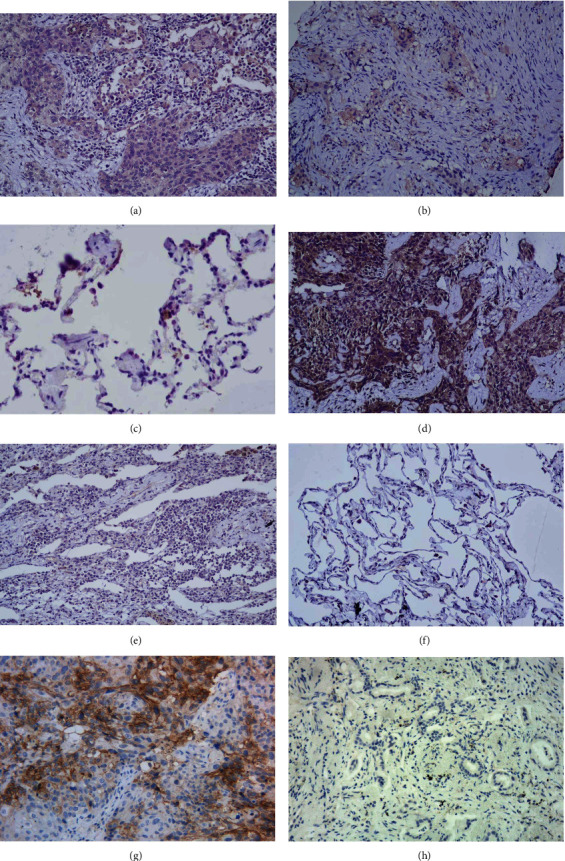
Representative immunohistochemical staining images of INSR, IRS-1, and PD-L1 in NSCLC tissues and adjacent normal tissues. (Original magnification: ×200). (a) Positive expression of INSR in NSCLC tissues. (b) Negative expression of INSR in NSCLC tissues. (c) Negative expression of INSR in adjacent normal tissues. (d) Positive expression of IRS-1 in NSCLC tissues. (e) Negative expression of IRS-1 in NSCLC tissues. (f) Negative expression of IRS-1 in adjacent normal tissues. (g) Positive expression of PD-L1 in NSCLC tissues. (h) Negative expression of PD-L1 in NSCLC tissues.

The expression of INSR and IRS-1 in 45 cases of nonsmall cell lung cancer and 30 cases of adjacent normal lung tissues was detected by immunohistochemical staining (Figure 1). The results showed that INSR and IRS-1 are expressed in both lung cancer and adjacent normal tissues. The expression of INSR was dramatically higher in 29 (64.4%) cases compared with 11 (36.7%) cases in adjacent normal tissues (*X*^2^ = 5.580, *P* = 0.018). IRS-1 expression was positive in 26 (57.8%) lung cancer tissues, which was significantly higher than 10 (33.3%) cases in adjacent tissues (*X*^2^ = 4.309, *P* < 0.05) (Table 1).

### 3.2. The Relationship between INSR, IRS-1 Expression, and Clinicopathological Features in NSCLC

The results of statistical analysis showed that the positive expression of INSR was correlated with the degree of pathological differentiation of NSCLC patients, and the difference was statistically significant (*P* = 0.031). There was no correlation with age, gender, pathological type, TNM stage, and lymph node metastasis status (*P* > 0.05). There was no significant correlation between the positive expression of IRS-1 and NSCLC patients' age, gender, pathological typing, degree of differentiation, TNM stage, and lymph node metastasis (*P* > 0.05) (Table 2).

### 3.3. The Relationship between PD-L1 Expression and Clinicopathological Features in NSCLC

There were 24 patients with positive expression, including 6 patients with high expression of PD-L1 (TPS ≥ 50%) and 18 patients with low expression of PD-L1 (1% ≤ TPS < 50%) (Figure 1). The results of statistical analysis showed that the positive expression of PD-L1 protein was correlated with lymph node metastasis (*P* = 0.028), while there was no significant correlation with gender, age, pathological type, TNM stage, and pathological differentiation degree of NSCLC patients (*P* > 0.05) (Table 3).

### 3.4. Correlation between INSR and PD-L1 Expressions in NSCLC

Spearman rank correlation was used to analyze the correlation between INSR, IRS-1, and PD-L1 expressions in nonsmall cell lung cancer. The results showed that there was no correlation between the expression of INSR and PD-L1 in nonsmall cell lung cancer (*r* = 0.143, *P* > 0.05) (Table 4). There was a positive correlation between the expression of IRS-1 and PD-L1 in nonsmall cell lung cancer (*r* = 0.373, *P* < 0.005) (Table 5).

## 4. Discussion

The reprogramming of tumor energy metabolism is considered to be one of the typical features of cancer, which endows tumor cells with the potential to continue to grow and proliferate in the tumor microenvironment (TME) with nutrient deficiency [18, 19]. As early as 1924, Ott Warburg first observed this abnormality of energy metabolism of tumor cells. Even in the case of sufficient oxygen supply, tumor cells still prefer to change their metabolic dependence from mitochondrial oxidative phosphorylation (OXPHOS) to glycolysis to generate energy [20]. After that, Zhou et al. [21] also found that drug-resistant cell lines have higher levels of aerobic glycolysis capacity, suggesting that there is a biochemical link between drug resistance and glycolysis. Oronsky et al. [22] further proposed that ATP produced by intracellular metabolism is the key determinant of chemoradiotherapy resistance. Controlling the metabolic process in vivo to limit ATP level can improve chemoradiotherapy sensitivity. In conclusion, energy metabolism reprogramming plays an important role in tumorigenesis and development. Therefore, targeted intervention on glycolysis has brought a new idea for tumor treatment, and the relevant studies have also confirmed the effectiveness of this treatment method [23]. Glucose transmembrane transport is considered as the rate limiting step of glycolysis [24]. In order to make up for the low efficiency of glycolysis energy production, tumor cells tend to ingest glucose at a higher rate, which is mediated by glucose transporters (GLUTs). As a transmembrane protein, gluts are widely distributed in human tissues. Up to now, 14 glut subtypes (Glut 1-14) have been found. Gluts, especially Glut1/3, have been observed to be highly expressed in a variety of different types of tumors, such as lung cancer and pancreatic cancer. Its overexpression not only significantly enhanced glucose uptake but also showed a close relationship with the poor tumor prognosis [24–26]. Small molecule inhibitors targeting gluts, including phloretin, WZB117, and STF-31, had also shown good effects in antitumor therapy [27, 28]. Studies have shown that phosphoinositide-3 kinase (PI3K/AKT) and other pathways are central regulators of glycolysis, tumor metabolism, and cancer cell proliferation [29]. In tumor cells, PI3K/AKT pathway can induce the expression of gluts and enhance the glycolysis process [30–33]. In addition, PI3K-Akt pathway is also a classical insulin downstream signaling pathway. In the insulin signaling pathway, when insulin binds to the INSRs on the cell membrane, it enhances the binding affinity between the receptor and IRS protein, causing the phosphorylation of IRSs to be activated. Then, IRSs can activate this pathway by recruiting and activating PI3K. Therefore, we have reason to believe that INSR and IRS-1 are involved in the process of glycolysis metabolism and play an important role in maintaining the survival and proliferation of tumor cells. They are expected to become potential targets for antitumor therapy. Our studies also showed that the positive rate of INSR and IRS-1 protein expression in NSCLC tissues was significantly higher than that in adjacent normal tissues (*P* < 0.05), suggesting that the expression of they may be involved in the occurrence and development of nonsmall cell lung cancer, which further verified our hypothesis. Heckl et al. have shown that PI3K-Akt pathway activation by insulin serves as an oncogenic driver for the development of NSCLC [34], which supports the investigation of insulin signaling pathways. Furthermore, insulin also acts to induce PD-L1 in pancreatic cancer, and while its role in NSCLC is not directly experimentally verified, it suggests a need to investigate insulin signaling and PD-L1 in NSCLC [35]. In the present study, tissue differentiation was significantly associated with INSR, while lymph node metastasis was linked to PD-L1.

PD-L1 is a recognized immunosuppressive molecule. Tumor cells can negatively regulate the activity of T cells by increasing the expression of PD-L1 on the cell surface, so as to achieve the occurrence of immune escape. With the approval of immune checkpoint inhibitor, immunotherapy against PD-L1 has shown good therapeutic effect in nonsmall cell lung cancer, but its immune-related adverse toxicity cannot be ignored. Therefore, looking for new targets to specifically regulate PD-L1 expression will help to more accurately block tumor-related PD-1/PD-L1 pathway and reduce adverse immune reactions. Previous studies have shown that PI3K/AKT pathway activation can promote PD-L1 expression by increasing exogenous signals or reducing the expression of negative regulatory factors such as PTEN [36]. Zhao et al. [37] confirmed that PD-1/PD-L1 blockade may inhibit the apoptosis of CD8+ T cells in gastrointestinal stromal tumors (GIST) by regulating PI3K/Akt/mTOR pathway. Stutvoet et al. [38] showed that inhibition of MAPK pathway can regulate EGF- and IFN-induced PD-L1 expression in lung adenocarcinoma. These results all suggest that the expression of PD-L1 can be regulated by PI3K/AKT and MAPK signaling pathway. These two pathways happen to be the most important downstream pathways of INSR and IRS-1. Whether they can regulate the abnormal expression of PD-L1 through these pathways has not been clarified yet. IRS proteins play a key role in tumor metabolism by regulating signaling of INSR. However, IRS-1 is also ubiquitously expressed in cancer cells. The differential expressions of IRS-1 versus IRS-2 may regulate drug responses. Further studies are essential to clarify complex signaling mechanisms [39]. Therefore, we used spearman correlation analysis to explore the relationships between them. The result showed that there is a positive correlation between the expression of IRS-1 and PD-L in NSCLC tissues (*r* = 0.373). Therefore, we speculate that IRS-1 may participate in the expression regulation of PD-L1 and mediate the immune escape process of NSCLC. However, the specific regulatory mechanism between IRS-1 and PD-L1 needs to be further explored in the follow-up study. At the same time, clinical studies with larger sample sizes are essential to understand the specific disease characteristics and prognosis concerning this regulatory axis. The present study utilized IHC alone, and studies at gene, mRNA, and protein expression levels are essential.

## 5. Conclusion

INSR and IRS-1 are both overexpression in lung cancer tissues, and IRS-1 expression had a significant positive correlation with PD-L1 expression. The expression of IRS-1 may further mediate the occurrence of tumor immune escape. Therefore, a better understanding of the regulatory mechanism of IRS-1 on PD-L1 expression will help to find new targets for NSCLC immunotherapy and provide new clinical ideas for immunosuppressive therapy.

## Figures and Tables

**Table 1:** Difference of expression of INSR and IRS-1 in lung cancer and normal lung tissue.

Variable	Total	INSR (%)		*X* ^2^	*P*	IRS-1 (%)		*X* ^2^	*P*
		+	-			+	-		
Tumor tissues	45	29 (64.4)	16 (35.6)	5.580	0.018	26 (57.8)	19 (42.2)	4.309	0.038
Adjacent tissues	30	11 (36.7)	19 (63.3)			10 (33.3)	20 (66.7)		

**Table 2:** The relationship between INSR, IRS-1 expression, and clinicopathological features in NSCLC.

Clinicopathological features		Total	INSR (%)		*P*	IRS-1 (%)		*P*
			+	-		+	-	
Gender	Man	26	17 (65.4)	9 (34.6)	0.878	14 (33.3)	12 (33.3)	0.532
	Female	19	12 (63.2)	7 (36.8)		12 (63.2)	7 (36.8)	

Age	<60	20	13 (65.0)	7 (35.0)	0.944	12 (60.0)	8 (40.0)	0.787
	≥60	25	16 (64.0)	9 (36.0)		14 (56.0)	11 (44.0)	

Pathological type	Squamous carcinoma	12	7 (58.3)	5 (41.7)	0.606	5 (41.7)	7 (58.3)	0.187
	Adenocarcinoma	33	22 (66.7)	11 (33.3)		21 (63.6)	12 (36.4)	

TNM stage	I + II	19	10 (52.6)	9 (47.4)	0.157	12 (63.2)	7 (36.8)	0.532
	III + IV	26	19 (73.1)	7 (26.9)		14 (53.8)	12 (46.2)	

Tissue differentiation	Moderately-well	27	14 (51.9)	13 (48.1)	0.031	16 (59.3)	11 (40.7)	0.805
	Poorly	18	15 (83.3)	3 (16.7)		10 (55.6)	8 (44.4)	

Lymph node metastasis	No	18	11 (61.1)	7 (38.9)	0.712	10 (55.6)	8 (44.4)	0.309
	Yes	27	15 (55.6)	12 (44.4)		19 (70.4)	8 (29.6)	

**Table 3:** The relationship between PD-L1 expression and clinicopathological features in NSCLC.

Clinicopathological features		Total	PD-L1 (%)		*P*
			+	-	
Gender	Man	26	14 (53.8)	12 (46.2)	0.936
	Female	19	10 (52.6)	9 (47.4)	

Age	<60	20	12 (60.0)	8 (40.0)	0.423
	≥60	25	12 (48.0)	13 (52.0)	

Pathological type	Squamous carcinoma	12	8 (66.7)	4 (33.3)	0.280
	Adenocarcinoma	33	16 (48.5)	17 (51.5)	

TNM stage	I + II	19	7 (36.8)	12 (63.2)	0.058
	III + IV	26	17 (65.4)	9 (34.6)	

Tissue differentiation	Moderately-well	27	14 (51.9)	13 (48.1)	0.807
	Poorly	18	10 (55.6)	8 (44.4)	

Lymph node metastasis	No	18	6 (33.3)	12 (66.7)	0.028
	Yes	27	18 (66.7)	9 (33.3)	

**Table 4:** Correlation between INSR and PD-L1 expression in NSCLC.

INSR	PD-L1		*n*	*r*	*P*
	+	-			
+	17	12	29	0.143	0.350
-	7	9	16		
*n*	24	21	45		

**Table 5:** Correlation between IRS-1 and PD-L1 expression in NSCLC.

IRS-1	PD-L1		*n*	*r*	*P*
	+	-			
+	18	8	26	0.373	0.012
-	6	13	19		
*n*	24	21	45		

## Data Availability

The study data presented may be made available from the corresponding author upon reasonable request.
